# Digital reminiscence app co‐created by people living with dementia and carers: Usability and eye gaze analysis

**DOI:** 10.1111/hex.13251

**Published:** 2021-06-15

**Authors:** Kyle Boyd, Raymond Bond, Assumpta Ryan, Deborah Goode, Maurice Mulvenna

**Affiliations:** ^1^ Ulster University Belfast UK; ^2^ Ulster University Newtownabbey UK; ^3^ Ulster University Londonderry UK

**Keywords:** apps, dementia, digital interventions, eye‐gaze, healthcare, human–computer interaction, reminiscence, usability, user interfaces

## Abstract

**Background:**

This research reports on a pilot study that examined the usability of a reminiscence app called ‘InspireD’ using eye tracking technology. The InspireD app is a bespoke digital intervention aimed at supporting personalized reminiscence for people living with dementia and their carers. The app was developed and refined in two co‐creation workshops and subsequently tested in a third workshop using eye tracking technology.

**Intervention:**

Eye tracking was used to gain insight into the user's cognition since our previous work showed that the think‐aloud protocol can add to cognitive burden for people living with dementia while also making the test more unnatural.

**Results:**

Results showed that there were no barriers to using a wearable eye tracker in this setting and participants were able to use the reminiscence app freely. However, some tasks required prompts from the observer when difficulties arose. While prompts are not normally used in usability testing (as some argue the prompting defeats the purpose of testing), we used ‘prompt frequency’ as a proxy for measuring the intuitiveness of the task. There was a correlation between task completion rates and prompt frequency. Results also showed that people living with dementia had fewer gaze fixations when compared to their carers. Carers had greater fixation and saccadic frequencies when compared to people living with dementia. This perhaps indicates that people living with dementia take more time to scan and consume information on an app. A number of identified usability issues are also discussed in the paper.

**Patient or Public Contribution:**

The study presents findings from three workshops which looked at user needs analysis, feedback and an eye tracking usability test combined involving 14 participants, 9 of whom were people living with dementia and the remaining 5 were carers.

## 1 INTRODUCTION

Dementia is a progressive condition which currently has no cure. The World Health Organization estimates that there are currently 50 million people living with dementia, with 10 million new cases every year.^1^ In the UK, there are an estimated 850 000 people living with dementia.^2^ Dementia impacts memory, concentration and judgement and can affect how a person goes about their activities of daily living, leaving people with the condition feeling isolated and lonely. In the UK, an estimated 700 000 family and friends are caring for a person living with dementia.^3^ Caring for a family member with dementia can be a very rewarding experience but it can also impact physical and psychological well‐being. The UK government considers new treatments for dementia as a priority and recognizes that technology for digital interventions has a role to play in supporting people living with dementia and their families.

Technology can be used to remind people living with dementia to take medication and to set calendar clocks to track their day. Locator devices can help people living with dementia to avoid the stress and anxiety associated with getting lost and telecare systems can help people stay safe. Digitally, video chat, social media, puzzles and games can all help to attenuate loneliness and maintain cognitive function.

With the expansion of touch screen device availability, downloadable apps and the importance of user‐centred design (UCD), people living with dementia and their carers (often a spouse or family member) have an important role to play when it comes to the design of digital health technologies. Research, innovation and digital interventions that provide optimal user experiences (UXs) can provide immediate support to transform the care and lives of people living with dementia. Recently, Hung et al^4^ undertook a scoping review to summarize the current knowledge of the impact of touchscreen interventions for people living with dementia. In all, they found 17 articles all of which looked at (a) increasing a person's engagement, (b) decreasing responsive behaviours (responsive behaviours refer to actions or gestures shown by a person living with dementia to respond to unmet needs or something negative and frustrating) and (c) positive effects on quality of life.

Nine studies found improvement in overall engagement with one study finding increased duration of engagement with touchscreen reminiscing app therapy. Four studies found decreases in responsive behaviours that were associated with iPad use as a non‐pharmacological intervention.^5^ The review provides useful insights into the use of touchscreen devices and the impact they can have. However, further research is needed to explore the co‐creation of apps involving people living with dementia, their families and health‐care staff. The mechanics of subtle touch (tapping, swiping, releasing) can be challenging and frustrating; hence, usability testing is necessary and useful.^6^

Goodall et al^7^ also conducted a systematic review of the use of technology to create individualized meaningful activities for people living with dementia. The review highlighted that by developing technology that is user‐friendly, user conscious and with the direct involvement of people living with dementia (which has been done with InspireD ^8^), the right balance of support and empowerment can be changed for everyone involved, patients, caregivers and family alike as they can all feel valued.^9^, ^10^ By looking at media from the past, they can engage not just with the past but with the present.^11^

In view of the current research involving people living with dementia and eye tracking, it appears that eye tracking can be used as a diagnostic tool. For example, work by Crawford et al^12^ reports that eye gaze data could be used as a diagnostic tool for mild cognitive impairment (MCI) or dementia. They studied the eye movements of a sample of 202 participants (42 living with dementia, 65 with MCI and 95 control participants). The anti‐saccade task (AST), where participants are required to make a saccadic eye movement away from a target, was used given that there is an emerging consensus that it provides a sensitive test of cognitive impairment. Findings revealed an overall increase in the frequency of AST for those with dementia and MCI. This highlights that eye movements recorded in eye tracking have the potential to help diagnose cognitive impairments. While this work is very interesting, eye tracking is typically used in one of two ways: (a) to allow a user to interact with a computer using their eye gaze as opposed to using a computer mouse and (b) for a user experience specialist to understand and gain insight into the visual processing of a user as they interact with a system. It is this latter purpose that this paper focussed on but with exploratory analysis to compare eye gaze behaviour between people living with dementia and their carers (carers who do not have a dementia diagnosis). Using eye tracking in usability tests can provide us with additional information about how the person living with dementia views the app. Eye tracking gives us this insight because what we look at—generally correlates with what we are thinking about. And given that the ‘think‐aloud’ method can be challenging as concurrently doing a task (interacting with an app) and verbalizing your thoughts induces additional cognitive burden,^13^ perhaps eye tracking is a useful method for gaining insight into the user experience of a person living with dementia.

### 1.1 Reminiscence

Activities such as reminiscence which stimulate memories for people living with dementia have been well documented.^14^ Butler^15^ first coined the phrase ‘Life Review’ and suggested that people in later years (particularly older adults) became more involved in a process of ‘looking back’ to identify, reflect, resolve or cherish memories from the past. Reminiscence is one of the most common non‐pharmacological interventions (NPI) for people living with dementia. Reminiscence NPI has been shown to have benefits related to the social, mental and emotional well‐being of participants.^8^ Reminiscence works by giving cognitive prompts using images, videos and music to evoke memories from the past that can influence mood and stimulate conversations with others. Historically, this was done through analogue methods such as the use of life story photo albums.

Westerhof and Bohlmeijer's^16^ review is testament to the work that has been completed over this period. The CIRCA system was developed which allowed people living with dementia and caregivers to explore stimuli like images and videos on a touchscreen.^17^ Fleischmann^18^ developed a personalized computerized system with a ‘front end’ interface for people living with dementia and a ‘back end’ interface for carers and family members to update the system. ‘Memory matters’ was an iPad reminiscence game where the researchers evaluated its efficacy. Of the sample tested (n = 80), the results showed that social interaction improved.^19^ Caros et al^20^ created a reminiscence chatbot that uses artificial intelligence (AI) technology to drive communication and inclusivity by using a combination of photos and questions. They coordinated a usability study and people with mild cognitive impairment found the system challenging but entertaining. This shows that using technology for reminiscence has potential, and it is worth further exploration. Nevertheless, to be effective, technology must meet user need and therefore usability testing is imperative. Previous work with an earlier prototype of the InspireD reminiscence app found that a personalized approach to reminiscence using a tablet app had the potential to have a positive impact on people living with dementia without negative consequences for their caregivers.^8^

The size, capacity and the low cost of ubiquitous devices and mobile tablet computers have made these technologies an attractive option for designing and delivering reminiscence. Moreover, the intuitive interaction that touch screens afford makes them more user‐friendly for people with lower levels of computer literacy.^21^ The intuitive interactions of touch screens are due to the ‘affordance’ of the device and its interface. Affordance is a property or feature of an object which presents a prompt on what can be done with the object.^22^ Affordances are cues which help users interact with something on a digital interface. An example would be a button. Its shape, size and colour will indicate that it can interact with. A touch screen is more akin to real‐world physics when compared to a traditional keyboard and mouse and matches the mental model of novice users.

### 1.2 Research questions

This paper focuses on one digital intervention (the ‘InspireD’ app) which was designed to support reminiscence for people living with dementia and their carers. The aim of this study was to investigate how users perceive the value of the InspireD app in terms of its usability and to make improvements to the app based on user feedback. We sought to answer the following research questions:

1. What are the usability issues and challenges when people living with dementia and their carers interact with the InspireD reminiscence app?
2. What is the correlation between task completion rates and the frequency of prompting? (frequency of prompting is the number of times the observer helped/prompt the user during a task)
3. What differences exist in usability metrics between people living with dementia and their carers when using the InspireD app?

## 2 METHODOLOGY

The study used a mixed‐methods approach incorporating quantitative and qualitative data. Participants were recruited through Dementia NI, a membership organization specifically for people living with dementia. The inclusion criteria specified that people living with dementia and their carers had to be 18 years or older, able to understand written and verbal information and to give informed consent. The inclusion criteria also specified that all participants needed to be able and willing to engage in the co‐creation, testing and evaluation of the reminiscence app. For this reason, only individuals with mild‐to‐moderate dementia were recruited to the study. Previous experience with app usage was not an inclusion criterion as the app was being designed to meet the needs of both novice and experienced users.

Ethical approval to conduct the study was granted by the institution where the researchers were based. All participants were provided with information leaflets about the study and signed a consent form prior to data collection.

### 2.1 Co‐creation app design

The principle ‘Nothing About Us Without Us’ is embedded in the global movement towards the active involvement of persons with disabilities in the planning of strategies and policies that affect their lives, and underpins the Convention on the Rights of Persons with Disabilities.^23^ As society moves to be more inclusive and the use of ubiquitous apps increases in popularity, it is imperative that everyone can use the technology irrespective of their physical or cognitive abilities and to test with intended users. The co‐creators helped to decide the overall aesthetic of the app including icons, typography as well as the usability of gestures and interactions of the app. This is detailed in Section 3.1.

### 2.2 The inspired reminiscence app

The InspireD app was designed by and for people living with dementia to enable them and their carers to select and store images, video and audio with notes to create a bespoke reminiscence experience.

The app builds on previous research and has been designed to support a joint reminiscence experience for people living with dementia and their carers. Our previous research found that in addition to joint reminiscence, people living with dementia also used the app independently at a time of their choice. Indeed, people living with dementia had more interactions with the app in comparison to carers.^24^

**FIGURE 1:**
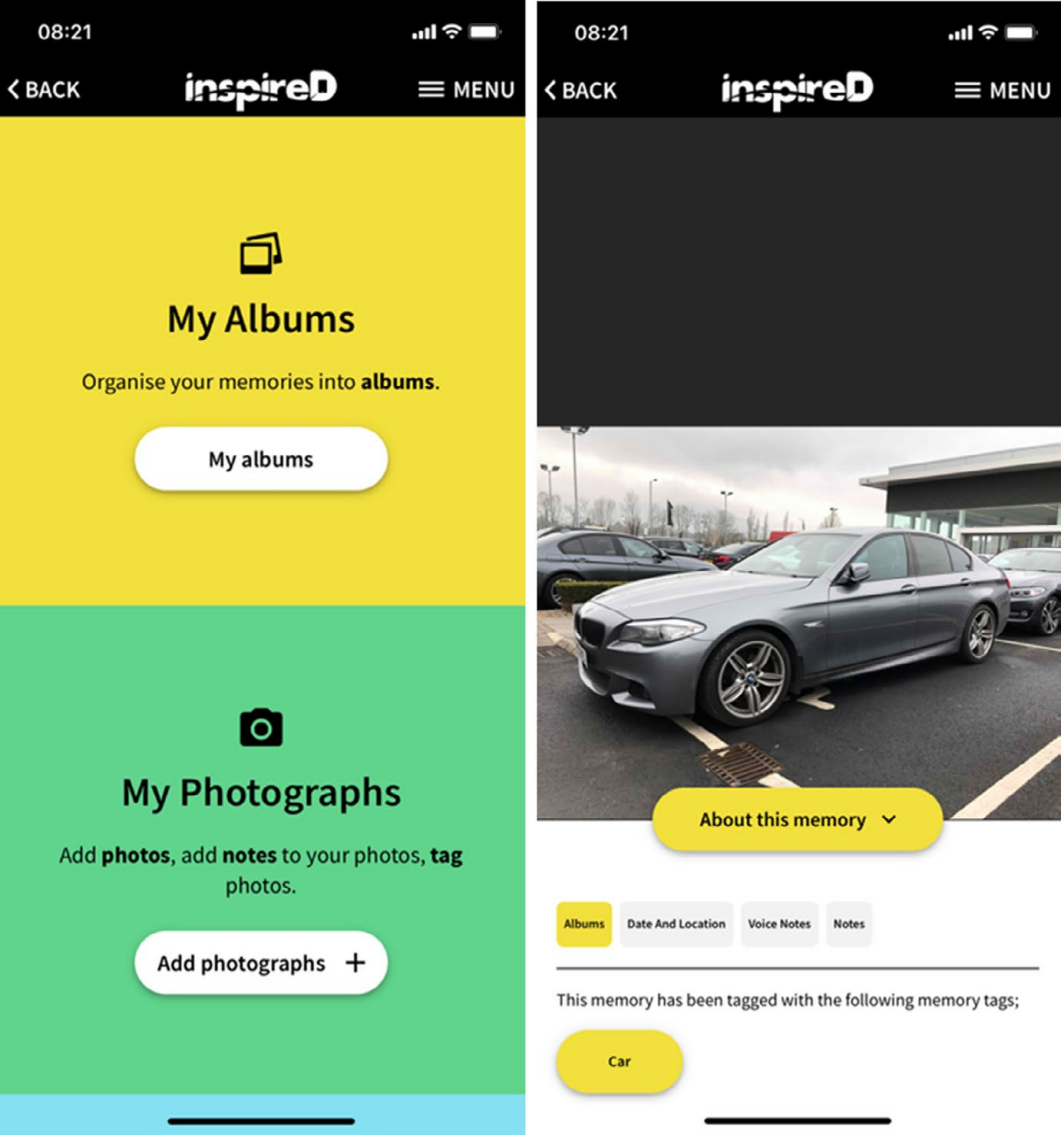
This is a sample of the user interfaces from the InspireD Reminiscence application. To the left shows the main dashboard and to the right is one of the picture detail screens

The app is minimal and uses clear and bold colours to compartmentalize the user interface. The user interface uses a mix of typography and iconography to guide participants through the app and to solidify mental models of how the app works. The app uses a step‐by‐step linear approach for completing complex tasks such as ‘uploading photos’ to maintain a lower demand on the user's cognitive load, that is the used amount of working memory resources. Figure 1 shows a screen image of the InspireD mobile application. This prototype was created using Invision, a web‐based prototyping tool that allows for the creation of interactive user experiences. The carer has access to the app so they can help the person with dementia if they have any issues accessing the content. The application also allows participants to restore deleted media and to create backups of images and notes to cloud‐based services such as Apple iCloud or Google. The researchers are also developing a website http://www.theinspiredapp.com which will contain additional resources and an explanatory video to assist users.

### 2.3 Workshops

Data were collected over three workshops which took place over a four‐month period between December 2019 and February 2020.

Workshops 1 and 2 (which were for user needs analysis and feedback) took place at the Dementia NI (a Northern Ireland dementia charity) group empowerment meetings. Preparation for working with people living with dementia was given to the app developers to ensure understanding of some of the issues around clear communication and taking time to listen and wait for responses. Involving the app developers in this process ensures an empathy‐centred design approach.

Eight participants attended Workshop 1, six people living with dementia, one volunteer and the Dementia NI empowerment officer. Three members of the app development team and one member of the research team were also present. The workshop lasted around one hour. The purpose of Workshop 1 was to try and tease out participants’ engagement with technology, use of apps and the coproduction of the app design.

Workshop 2 involved eight people living with dementia. One spouse and the empowerment officer attended this workshop in addition to three app developers and two members of the research team. This workshop was designed to demonstrate the prototype app on a large screen and on a tablet so that participants could get to hold the device and view it in a realistic format. Each step of the set‐up stage of the app was discussed and choices of layout, wording and usability agreed. The empowerment officer from Dementia NI who had a long‐established relationship with the group played a key role in facilitating this workshop as she knew the participants living with dementia very well and was able to encourage them to provide honest feedback on the app.

Workshop 3 (a usability test of the InspireD app) took place in the Living Lab at the university where the research was based. This is a purpose‐built facility for research in an environment, designed to resemble a living space with comfortable seating and a table. This is important for all research but is essential when working with people who are living with dementia. Public buildings were chosen specifically as they are required by law to be accessible for those with disabilities ensuring participant inclusivity.^25^

Ten participants (five people living with dementia and their carers (family member/friend)) undertook the usability test. Participants comprised four females and six males. Three dyads were married, one was a father and daughter and one comprised two male friends.

To minimize anxiety, participants were accompanied to the Living Lab by the empowerment officer from Dementia NI, who facilitated the usability testing and provided support to participants. Participating dyads (person living with dementia and their carer) were tested in pairs one after another with the carer tested first. This may have led to some bias as the second participant had already heard the tasks once, before they took the test. However, this approach was justified as a further measure to ensure that any anxiety associated with the test was minimized for participants living with dementia. Permission was granted for the researchers to audio‐record the meetings and take notes.

### 2.4 Usability testing

Usability testing refers to the process of evaluating a product or service by testing it with representative users. Typically, during a test, participants will try to complete tasks while observers watch, listen and take notes. The goal of the test is to identify any usability problems, collect qualitative and quantitative data and determine the participant's satisfaction with the product.^26^ In order to run effective usability testing, the development of a repeatable test protocol, appropriate participant recruitment, analysis and reporting is required.

### 2.5 Eye tracking

**FIGURE 2:**
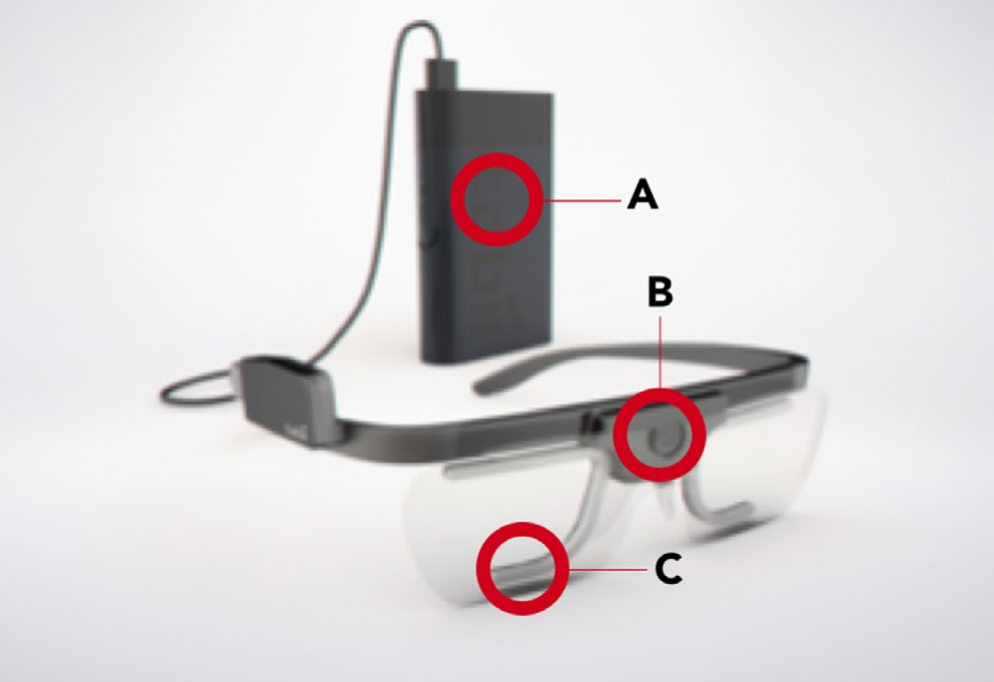
The eye tracking glasses which were used to record participants. These are the Tobii Glasses 2 wearable eye tracker. (A) Recording storage, (B) scene HD camera, (C) infrared cameras (Tobii Technology / CC BY (https://creativecommons.org/licenses/by/3.0)

Eye tracking is used to acquire eye movement patterns. The system collects two points of data: (a) a fixation—when an eye is resting or ‘fixated’ on an object, and (b) a saccade which is when the eye is moving from one point to another. When this raw eye gaze data are collected, it is possible to represent these features as heat and gaze maps (Figure 2).

A scan path of a user's eye movements is called a gaze map. The higher density of an eye movement on that part of the screen shows a higher probability that the user has seen this part of the screen. Nevertheless, researchers can compute eye gaze metrics as opposed to using data visualization approaches. The use of eye gaze metrics was the preferred choice for this study as they allowed for statistical tests as opposed to subjective visual inspection of graphs. Typical eye gaze metrics include fixation frequencies, saccadic frequencies and fixation durations.

## 3 RESULTS

The following breakdown details what happened in each of the three workshops and the results. The first workshop comprised a user needs analysis to explore participants' understanding of apps and their design. The second workshop was used to elicit feedback on an early prototype of InspireD and the third was the usability test on the InspireD app itself.

Of the 14 participants, 10 participants took part in workshop 3, 6 in workshop 2 and 4 in workshop 1. Some participants attended all workshops (Table 1). Table 1 also includes information about study participants, including the ages of participants living with dementia as these details were made available to the researchers from Dementia NI. However, as the sample was too small to draw any inferences regarding age and user engagement with the app, this information was not collected from carers.

**TABLE 1:** Details of the participants who took part in the workshops

Participant name (pseudonym used)	Role	Age	Length of time since diagnosis	Workshops attended 1,2, 3
Polly	Person living with dementia	60	3 y	Workshop 3
Tom	Husband to Polly	Carer		Workshop 3
David	Person living with dementia	64	3 y	Workshop 1 Workshop 2 Workshop 3
Laura	Daughter to David	Carer		Workshop 3
Daniel	Person living with dementia	65	5 y	Workshop 1 Workshop 2 Workshop 3
Emma	Wife to Daniel	Carer		Workshop 3
John	Person living with dementia	69	7 y	Workshop 3
Bobby	Friend of Johns	Carer		Workshop 3
Mark	Person living with dementia	73	4 y	Workshop 3
Florence	Wife of Mark	Carer		Workshop 3
Gwen	Person living with dementia	76	2 y	Workshop 2
Harry	Person living with dementia	69	2 y	Workshop 1 Workshop 2
May	Person living with dementia	81	3½ y	Workshop 1 Workshop 2
Gordon	Person living with dementia	67	3 y	Workshop 2

Note

Shaded rows show how people living with dementia know one another.

### 3.1 Workshops 1 and 2

In Workshop 1, all participants had a mobile phone but there was a mixed response in relation to the ability to use the phone, from simple phone calls to more extensive use of music downloads and social media apps. Several of the participants living with dementia emphasized their love of music and the role it held in everyday life:

Alan (pseudonym) explained that he ‘…*likes listening to music. My sons send me music and I can get onto it and play it on my phone’*. Mark agreed ‘*I use the phone; my daughter puts music on {for me}. I couldn't put it on myself’*.

There was some discussion about the function of an app. Participants knew that the apps they routinely used took them to the pages where they could listen to music or read sports results but most needed clarification about what an app was and how it worked. Icons for commonly used apps such as Facebook and WhatsApp were recognized and used by most of the group who explained that these apps helped them to stay connected to family members. YouTube was also popular for watching videos and music.

When discussing reminiscing, there was a consensus that reminiscence usually occurred with a family member and centred around photographs and stories. John said ‘*we would look at photos and old video films. I like to see the family coming in and out. Time flies*’. Ruth agreed and said about photos ‘*The kids get them out and ask, Who's that? They think you were never young. You think Oh my goodness, how long ago was that?*’. Alan explained how his dementia has affected the way he reminisces now; ‘…*Photos are good for the memory too. I would listen to CDs a lot. I can't get used to films any more with the dementia. I cry my eyes out with the music as it brings back all the memories*’.

The session then developed into a series of co‐creation type decisions or choices about usability of icons, buttons, size and colour of text and the appropriate wording for descriptions. Each participant was asked for their opinion. All responses were clarified and noted by the app developers. This included familiarity with scrolling up and down, swiping and zooming in on a photo.

When asked about their views on the most important feature of a reminiscence app, participants identified music, photos and videos. Alan explained ‘…*something that is there for a moment but can be there for years [in a video]. I think as the kids get older it is nice to have them [the videos] to look at and keep*’. Nigel said for him it was music because like Alan*, ‘reading or watching a film is hard to concentrate on. Music would be the main thing. I have about 200 photos on my phone and every now and then I would look at them’*.

In Workshop 2, key issues were highlighted by the group including text size and font in addition to icon size and location. Although the app was being designed for people living with dementia and their carers, some participants felt uncomfortable with the word ‘carer’. This resulted in a decision to use the term ‘carer and/or family member’ on the final version of the app.

People living with dementia were able to use the prototype on a tablet provided by the app developers. This, together with the large screen projection of the app, enabled all participants to contribute to the design of the reminiscence app. Most participants welcomed the idea of the app. Keith said that it would be useful for photos and songs to ‘*get them organised and in a system’*. Two participants who were less comfortable with technology felt that they would be relying on family members to help them use the app.

### 3.2 Workshop 3: Usability testing results

This was a pilot study, and therefore, no statistical power analysis was used to model participant sample size. Within usability testing, the sample sizes of between 5 and 15 are deemed appropriate, with 5 participants yielding 80% of usability issues.^27^ However, this guide for sample sizes is not necessarily appropriate for usability tests that use quantitative data analysis.

The participants undertook a usability test of a prototyped reminiscence app (called ‘InspireD’). Each subject was asked to complete 10 short tasks (Table 2). Short tasks were chosen as opposed to conventional scenario‐based tasks given that the scenario‐based tasks require more working memory and recall; hence, we avoided adding additional cognitive overload. A Tobii wearable eye tracker was used to record the eye gaze fixations of each participant while they interacted with the prototype. Eye tracking was used to provide insight into the user's cognitive processes. Although think‐aloud protocol (TAP) is a commonly used usability test, this approach was not used as it can overload working memory and prior research suggested that TAP was not appropriate for people living with dementia.^13^

**TABLE 2:** The tasks that were completed by participants using InspireD during the usability test

Task No	Task to complete
1	Adding a new album Start app at profile screen Add a new album
2	View album ‘Holidays 1987' Start app at dashboard screen View album ‘Holidays 1987
3	Add a Photo (resume) Resume from current position in the app (Likely to be ‘Holidays 1987’ album) Add a photo to the ‘Holidays 1987’ album
4	Add a Video (resume) Resume from current position in the app (Likely to be ‘Holidays 1987’ album) Add a video to the ‘Holidays 1987’ album
5	Add a music file (resume) Resume from current position in the app (Likely to be ‘Holidays 1987’ album) Add a music file to the ‘Holidays 1987’ album
6	View the image of people in the canoe Start from photo grid (Album ‘Holidays 1987’) View image of people sitting in canoe
7	Flick through other photos Start from image of people sitting in canoe (Album ‘Holidays 1987’) Look through first 4 photos
8	View notes on this picture Start from image of people sitting in canoe (Album ‘Holidays 1987’) View notes that have been added to this picture
9	Add a note Start from image of people sitting in canoe (Album ‘Holidays 1987’) Add a note to this image
10	View ‘Grandkids' album (resume) Resume from current position in the app (Likely to be ‘Holidays 1987’ album) View ‘Grandkids’ album

**FIGURE 3:**
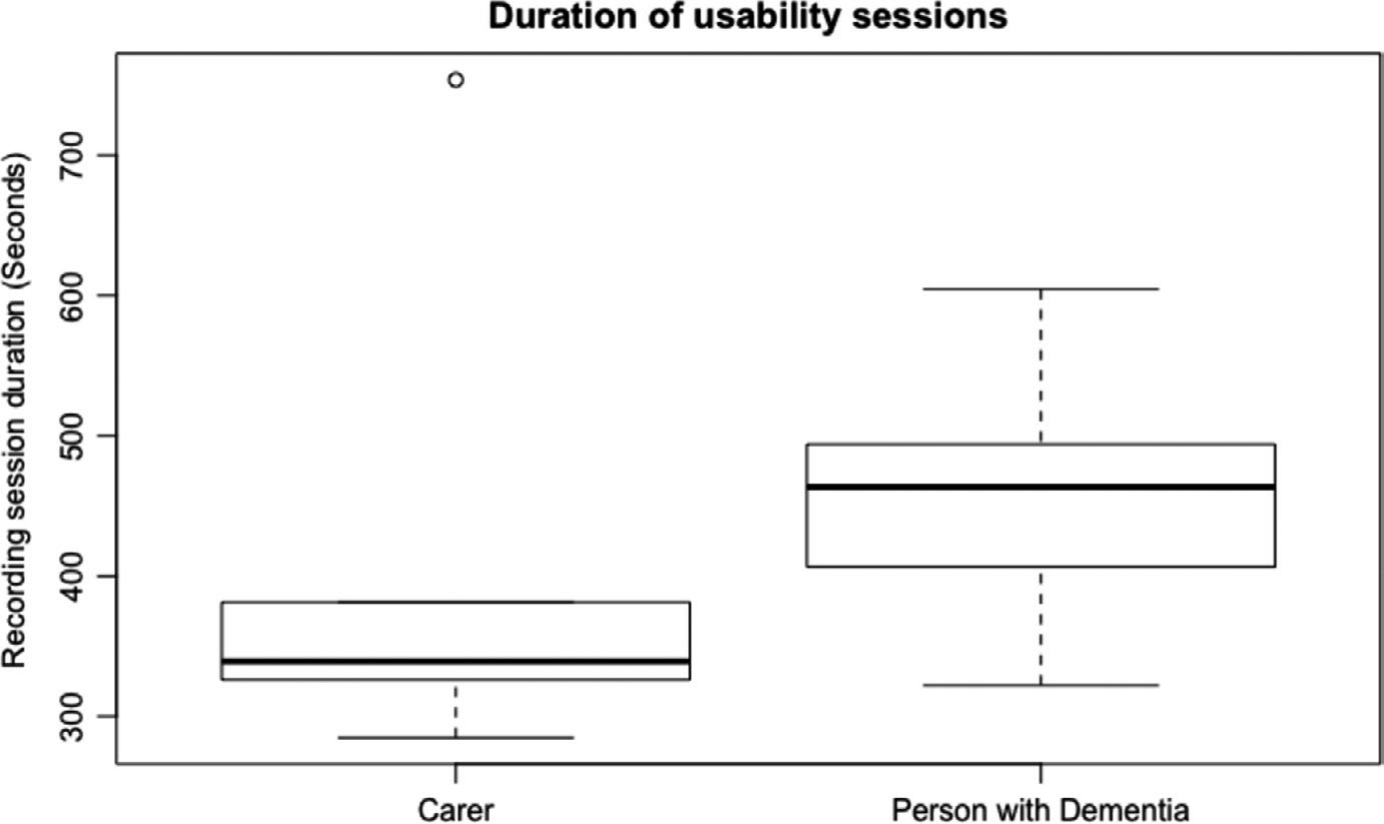
This boxplot shows the duration of the usability testing sessions (in seconds) for carers and people living with dementia. The two boxplots in the graph show the median average times (thick lines) for the carers and people living with dementia. The edges of the boxes represent the interquartile range (25th and 75th percentile) and the whiskers represent the minimum and maximum durations (except when there is an outlier—an outlier is a sample that is 1.5 times greater than the interquartile range). The boxplot shows that people with dementia need more time to interact with the app and to complete the tasks

**FIGURE 4:**
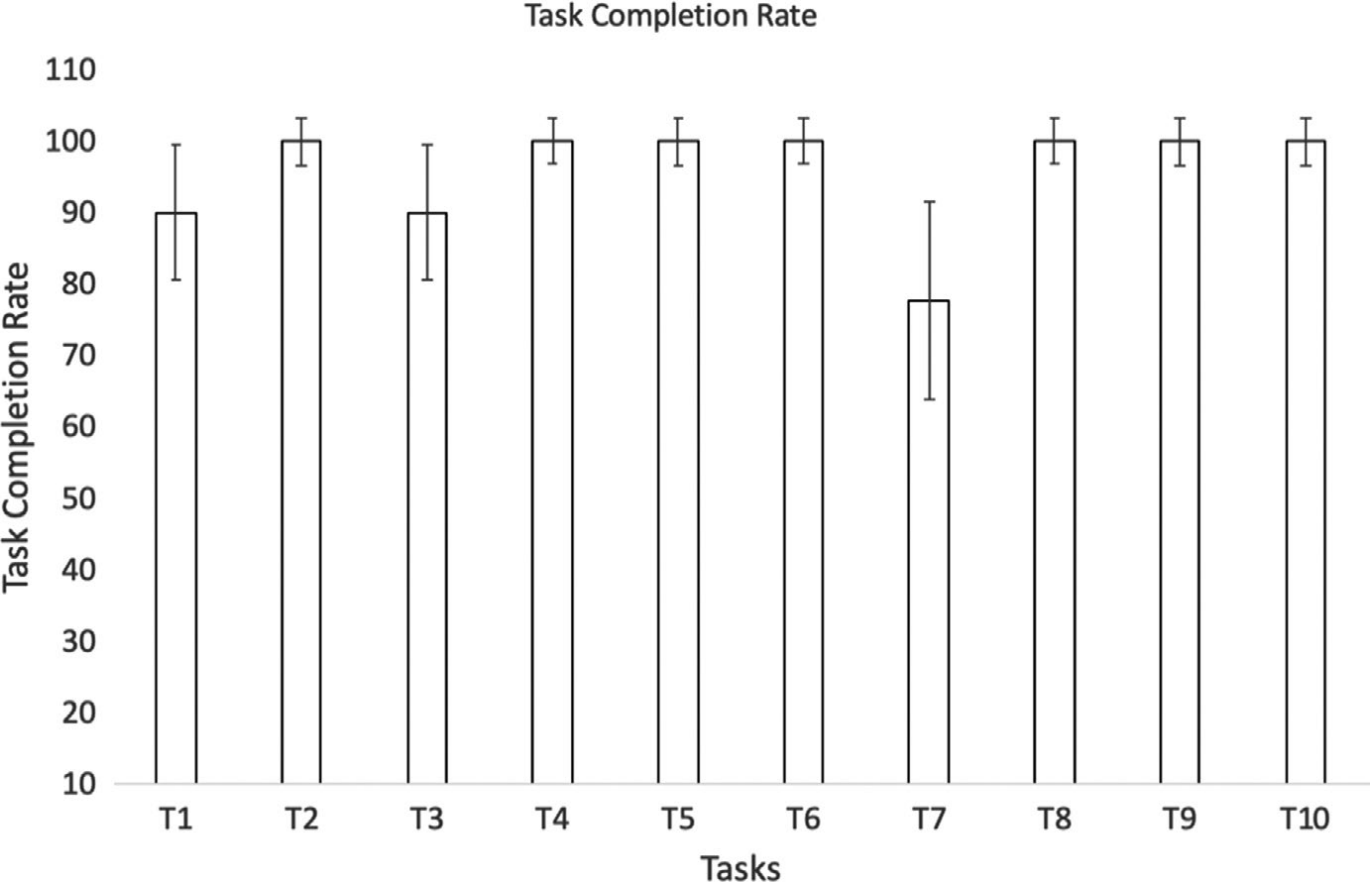
This bar chart shows the task completion rate for each of the 10 tasks (rate is normalized as a percentage, hence 100% = all subjects successfully completed the task, 0% = no subject completed the task). The error bars represent the ‘standard errors of the proportion’ (otherwise known as a 68% confidence interval). According to this metric, tasks 1, 3 and 7 were the most challenging

**FIGURE 5:**
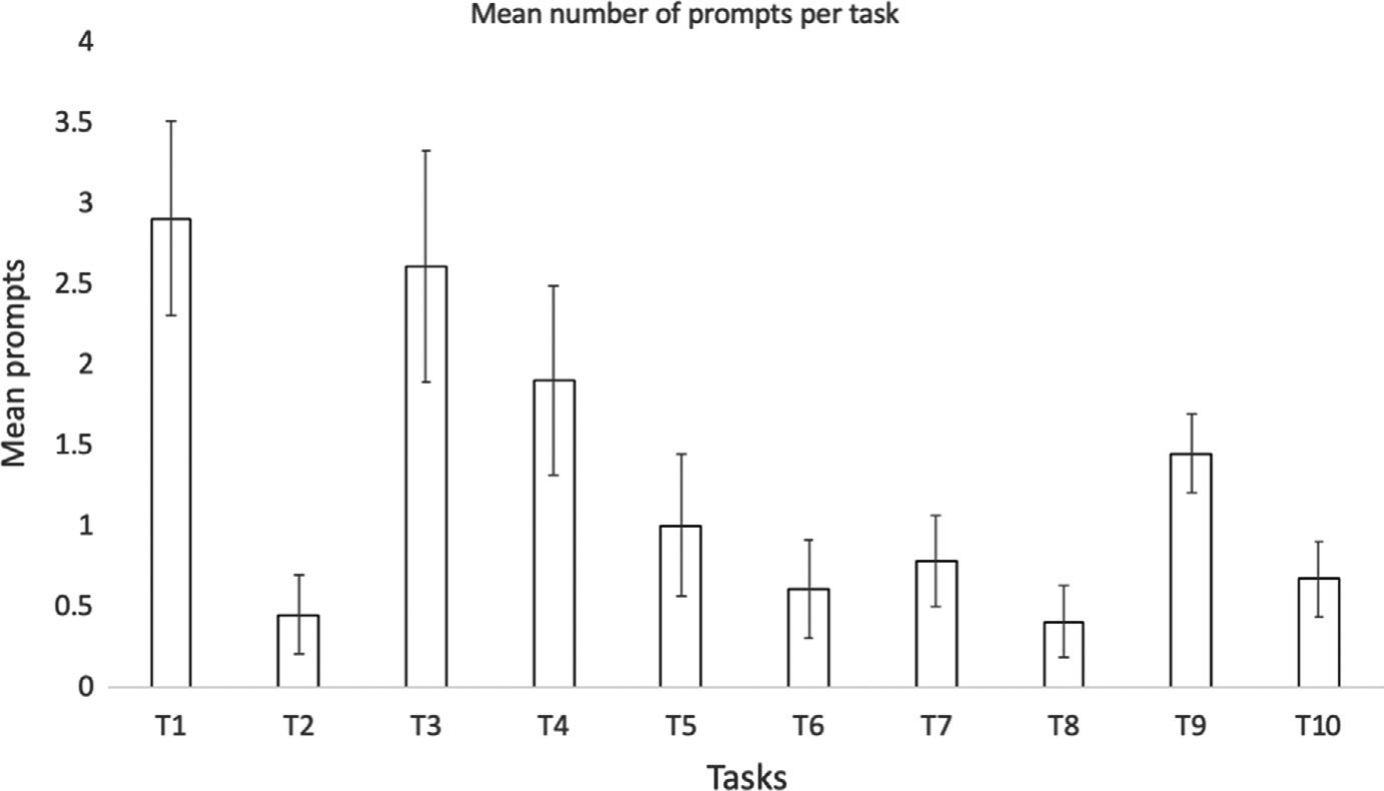
This bar chart shows the average number of prompts made by the researcher that subjects needed while doing each of the 10 tasks. The error bars represent the ‘standard errors of the mean’ (otherwise known as a 68% confidence interval). According to this metric, tasks 1, 3, 4 and 9 were the most challenging

**FIGURE 6:**
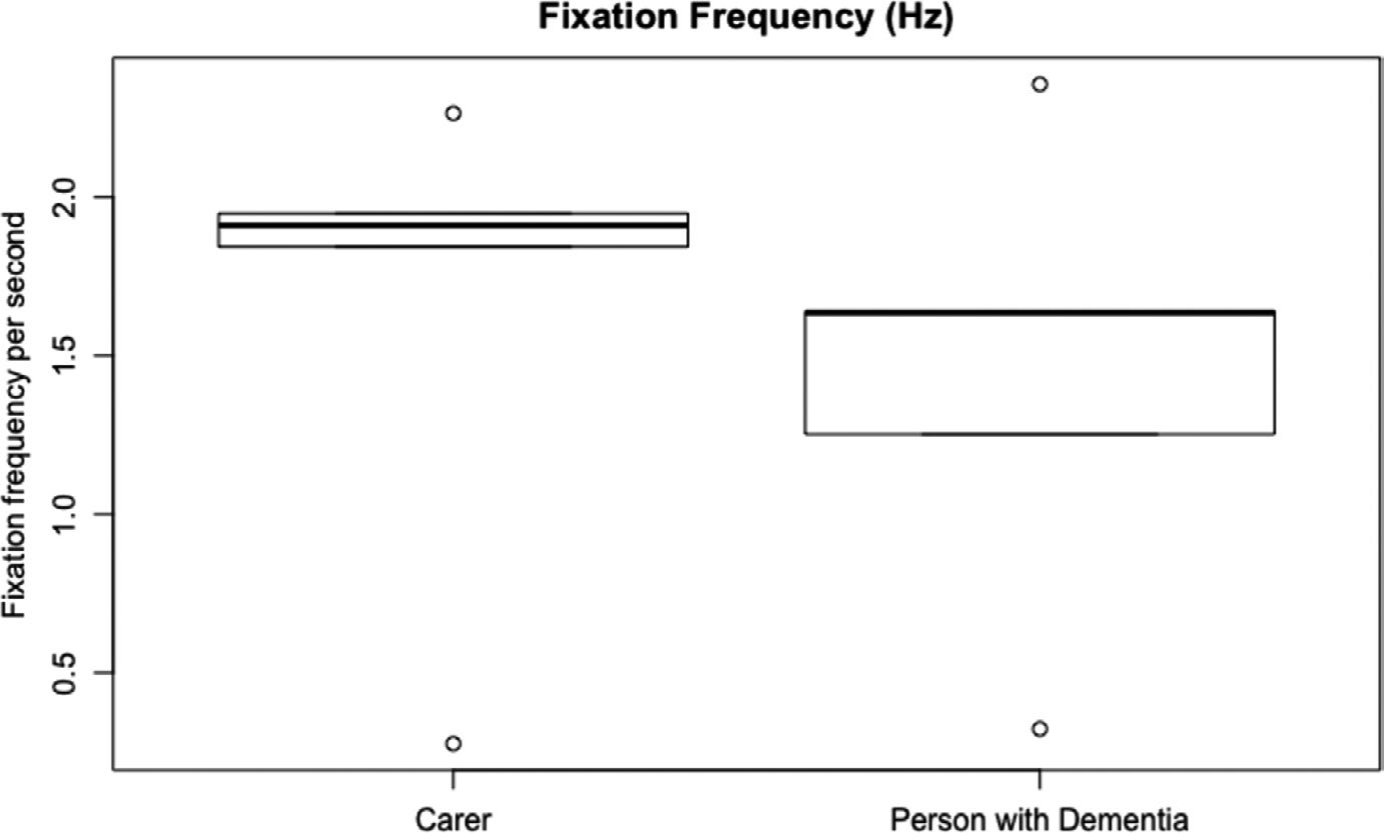
This boxplot shows the fixation frequency (ie number of fixations per second / Hz) for carers and people living with dementia. The two boxplots in the graph show the median average fixation frequency (thickest lines) for the carers and people living with dementia. The edges of the boxes represent the interquartile range (25th and 75th percentile) and the whiskers represent the minimum and maximum fixation frequency (except when there is an outlier—an outlier is a sample that is 1.5 times greater than the interquartile range)

**FIGURE 7:**
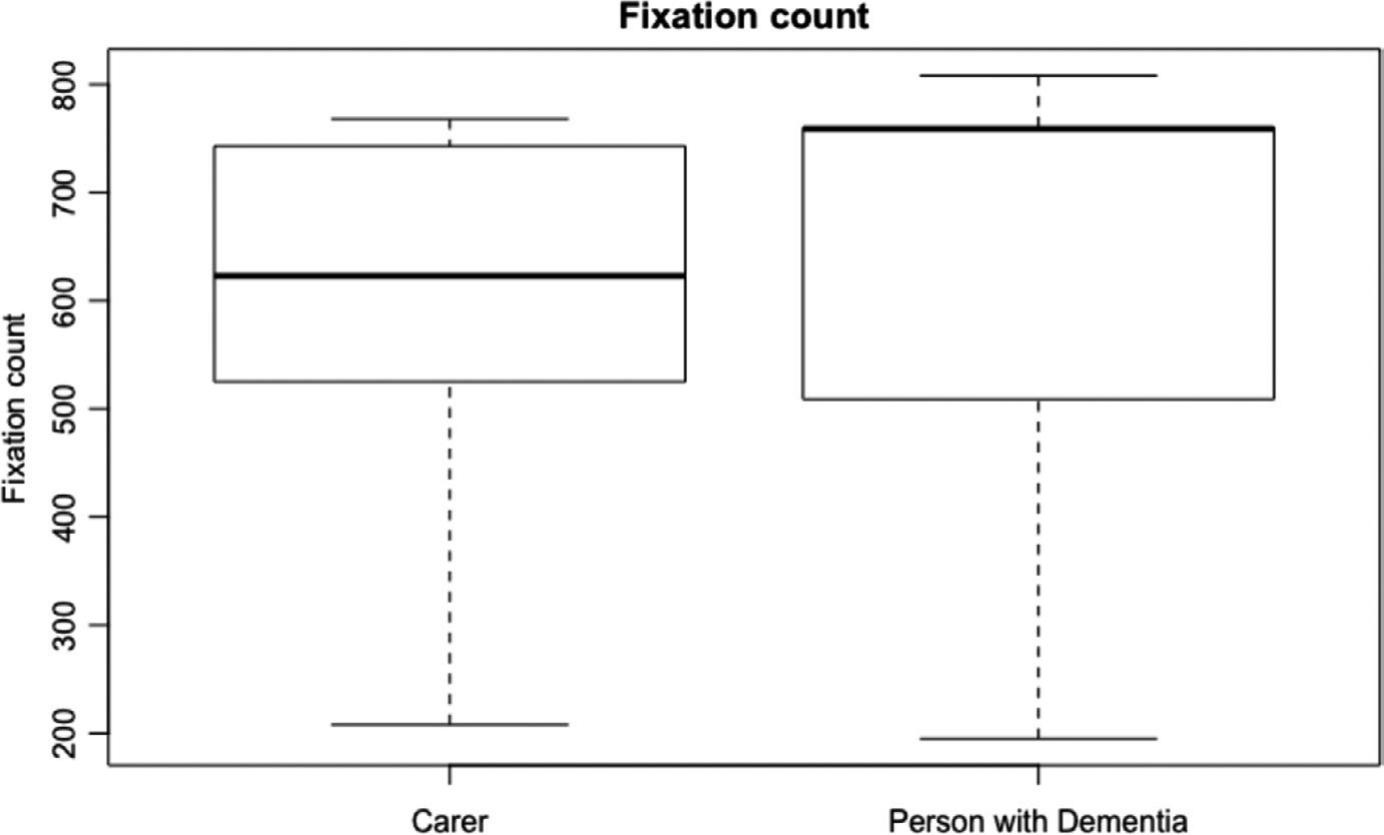
This boxplot shows the fixation count for carers and people living with dementia. The two boxplots in the graph show the median average fixation count (thickest lines) for the carers and people living with dementia. The edges of the boxes represent the interquartile range (25th and 75th percentile), and the whiskers represent the minimum and maximum fixation count

**FIGURE 8:**
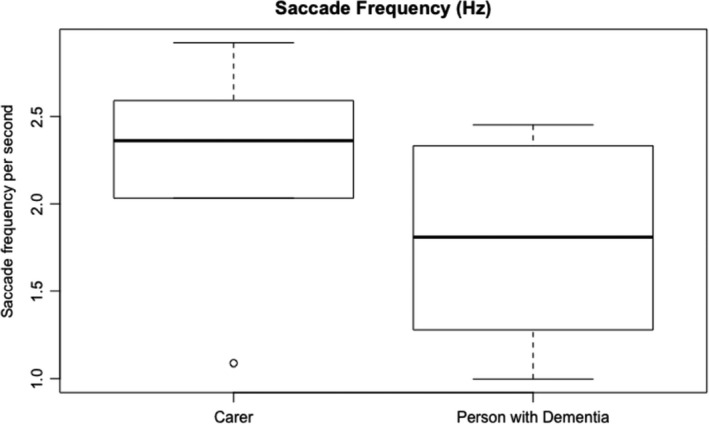
This boxplot shows the saccade frequency (number of saccades per second / Hz) for carers and people living with dementia. The two boxplots in the graph show the median average saccade frequency (thickest lines) for the carers and people living with dementia. The edges of the boxes represent the interquartile range (25th and 75th percentile) and the whiskers represent the minimum and maximum saccade frequency (except when there is an outlier—an outlier is a sample that is 1.5 times greater than the interquartile range)

**FIGURE 9:**
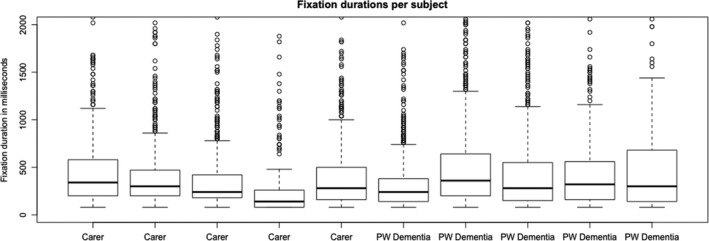
This boxplot shows the saccade frequency for each carer and each person living with dementia. The ten boxplots in the graph show the median average saccade frequency (thickest lines) for each carer and person living with dementia. The edges of the boxes represent the interquartile range (25th and 75th percentile) and the whiskers represent the minimum and maximum saccade frequency (except when there is an outlier—an outlier is a sample that is 1.5 times greater than the interquartile range)

Figure 3 shows the durations of the usability session indicating that people living with dementia spent more time completing the tasks. Task completion rates (Figure 4) were high with most tasks being completed by all subjects except for task 1 (90% ± 9.48), task 3 (90% ± 9.48) and task 7 (77.78% ± 13.86). Figure 5 shows the average number of prompts per task indicating that task 1 (2.9 ± 0.60), task 3 (2.6 ± 0.71), task 4 (1.9 ± 0.58) and task 9 (1.44 ± 0.24) required the highest number of prompts. This suggests that these tasks were less intuitive. There was some agreement between task completion times and number of prompts for tasks 1 and 3; however, tasks 4 and 9 achieved 100% task completion rates yet these tasks involved a number of prompts. Moreover, while task 7 involved a lower task completion rate, this task involved a lower number of prompts. This suggests that the ‘frequency of prompts’ is a useful metric that may provide additional insight into the usability of a system designed for people living with dementia. This hypothesis was supported given that there was a weak negative correlation between task completion rates and the frequency of prompting (*r* = −0.299; Figures 6, 7, 8, 9).

### 3.3 Usability issues

The usability issues that occurred among participants are presented in Table 3. The table also presents the frequency of each usability issue occurring.

**TABLE 3:** Main usability issues, the frequency they occurred and how they were resolved

Issue Number	Usability Issue	Frequency	How it was resolved
1	Limitations of the prototype itself got in the way of the UX for participants	10	Limitations of prototype application
2	Participants could not find the ‘add album’ button on the main dashboard and did not know what ‘album’ meant from the app perspective (perhaps a misplaced mental model)	7	The wording was changed to create album. To be clearer and more explicit
3	The ‘add memories’ button was not understood/seen	5	This button was changed to say ‘Add Photo, Video, Sound’ to be explicitly clear on functionality
4	The participant could not see the ‘next’ and ‘previous’ buttons that were superimposed on the photos. Participants used the ‘Back to albums’ button to view the next image in the sequence	3	Added more traditional arrows on media to indicate left and right movements. You can now also ‘swipe’ between media as you would expect on any smart device

## 4 DISCUSSION

The findings of this study tentatively suggest that eye gaze behaviour for people living with dementia may be different from that of people without dementia/carers. This hypothesis concurs with work that has been published which investigated the use of eye tracking as a diagnostic tool or biomarker for mild cognitive impairment and dementia.^12^ While this is an interesting hypothesis, we have discussed the specific findings in this paper in the following paragraphs. Firstly, we will revisit and discuss the research questions.

### 4.1 What are the usability issues when people living with dementia and carers interact with a reminiscence app?

In general, workshop feedback suggested that people living with dementia may need some explanation to understand what an app is and how it works, although they are familiar with mainstream apps (eg YouTube) and icons. Some of the usability challenges related to common problems for older participants such as position and size of text, icons and colour. Many of the participants had issues surrounding the text and understanding what certain terms meant with reference to the application. For example, when asked to add an album, one participant asked, ‘do you mean music?’ rather than a digital photo album. This shows that language and particularly copywriting should be considered for the intended users and those of a particular age.^28^ Another issue was that some of the participants with dementia could not identify the call to action buttons or buttons that would lead to completing tasks. Perhaps this was due to ‘cognitive hysteresis’ (tunnel vision) which can affect some people living with dementia.^29^ In order to remedy this issue, the call to actions may need a higher colour contrast and be of an appropriate aesthetic for people living with dementia.^30^, ^31^

Many issues that were found were not major; however, minor changes to the overall interface could make a big difference to the user experience. In general, it was felt that the input fields needed to be clearer and offer better ‘affordance’ to indicate that participants were required to input text.

The authors suggest that animation could be used to attract attention to the main call to action buttons which would amplify the visual hierarchy of the interface. This was particularly obvious for the ‘next’ and ‘previous’ swipe buttons for navigating through photos as many participants in this cohort did not know the ‘swipe gesture’ which is an interesting finding. The usability test resulted in many positive findings since most tasks had a high completion rate and with only a small number of prompts. After participants had used the app to complete the first number of tasks, they quickly adapted to the user flow making many of the subsequent tasks easier. From a user experience perspective, informal feedback from participants after the testing indicated that user satisfaction was positive throughout all participant interactions and the acceptability of the app was high given the high level of engagement by all participants. In order to help with more complex interactions, training may also be an appropriate support for participants.

### 4.2 What is the correlation between task completion rates and the frequency of prompting?

Many of the tasks (see Table 2 for reference) were completed apart from task 1 (90% ± 9.48), task 3 (90% ± 9.48) and task 7 (77.78% ± 13.86). These three tasks require users to interact with buttons (ie adding an album, photo and swiping through photos) which highlight the issues previously discussed.

From our results, tasks 1, 3, 4 and 9 needed the greatest number of prompts for them to be completed. Both tasks 4 and 9 achieved 100% completion rates. These tasks involved adding an album, adding a photo, adding a video and adding a note which are perhaps perceived as the most complex tasks on the InspireD app due to the number of steps required. Moreover, while task 7 achieved a lower task completion rate, this task involved fewer prompts. Hence, this suggests that the ‘frequency of prompts’ is a useful metric which provides additional insight into the usability of an interactive task. Therefore, there is a correlation between task completion rates and the frequency of prompts, the more prompts the better completion rate.

Indeed, prompting provided by a person or a computer can yield a positive user experience by providing context and communication for people living with dementia.^32^ Prompting can aid participants based on their cognitive impairment such as memory loss, attention span, reasoning and judgement and complex decision making.

### 4.3 What are the differences in usability metrics between metrics acquired from people living with dementia and carers, using a reminiscence app?

Regular usability metrics normally include success rate, time on task, error rate and the users’ subjective satisfaction. We found no differences in task completion rates between the two groups with both groups completing most tasks which is a testament to the usability of the app. There was a difference between the time on task with people living with dementia taking longer to complete tasks when compared to carers. This was shown during the more cognitively challenging tasks in the test. Due to people living with dementia taking more time to complete tasks, they exhibited more gaze fixations during the test which is understandable given that they took more time; however (see Figure 7), people living with dementia did have a lower number of saccades. This finding builds on the work by Wilcocksin et al^33^ and Molitor^34^ and others who propose eye tracking as a useful diagnostic biomarker in the assessment of dementia.

Future work on the development of the InspireD reminiscence app will focus on completing the build of the app and then releasing this on ORCHA (Organisation of the Review of Care and Health Apps) a health‐related app store which reviews the security and safety of health‐related apps. It is the research team's intention to run further usability studies on InspireD with the completed application in the future.

## 5 CONCLUSION

The paper reports the findings of a series of co‐creation workshops and a usability study on a prototype of the InspireD reminiscence app, designed to support people living with dementia and their carers. While the numbers tested were low, results showed that participants had issues with colour contrast and size of call to action buttons which they could not see. They also had issues with the copywriting highlighting the importance of using language which can be understood by its intended users.

During the data analysis of the usability test, the authors observed that facilitators prompted people living with dementia if they were struggling to complete a task. Some of the most complex tasks like adding a photo required the most prompts, while those tasks with lower completion rates had a fewer number of prompts. This suggests that ‘frequency of prompts’ could be used as an additional usability metric when studying the usability of interactive tasks with people living with dementia.

The study concluded that people living with dementia and their carers displayed some differences regarding usability metrics. People living with dementia had more gaze fixations given that some tasks took longer to complete but did show a lower number of saccades. This builds on work that supports the use of eye tracking as a diagnostic biomarker in the assessment of dementia.

## 6 POLICY AND PRACTICE

The findings of this study could have an impact on policy and practice for digital health dementia apps in the future. It has shown that eye gaze techniques when modified can be successfully employed to assess usability of apps for use by people living with dementia. This has significant practice and indeed policy implications, given that NHS and NI Dept of Health's PHA have invested in an online dementia app portal (https://apps4dementia.orcha.co.uk/) broadening out to apps for general health (https://apps4healthcareni.hscni.net/).

## 7 LIMITATIONS

It can be argued that having more participants may have added to the quality of data collection. Nevertheless, it was felt that the qualitative findings from the workshop provided a beneficial insight into the co‐creation of digital health apps and how to facilitate usability studies for people living with dementia. The facilitator who assisted with the usability study had a strong rapport with the participants, so it was natural to provide prompts. In future usability studies, an alternative may be to limit the prompting by using the echo or boomerang techniques, whereby questions are repeated but in a different tone to avoid prompting.^35^ However, prompt frequency was measured and can be used as a usability metric. Furthermore, perhaps this metric would be suitable for usability tests for people living with dementia, or a cognitive impairment.

## AUTHOR CONTRIBUTIONS

Kyle Boyd reviewed the usability testing recordings, collated the issues found and how to improve them and contributed to the main content of the paper. Raymond Bond conducted the necessary data analysis of the usability testing results. Assumpta Ryan was present at the workshop sessions and contributed to the write‐up. Deborah Goode was present at the workshops and completed the majority of the write up for these. Maurice Mulvenna conducted and overall edited the paper during write‐up.

## ETHICAL STATEMENT

Following ethical approval by the university conducting the study, the app was co‐designed by people living with dementia in collaboration with the research team and the software company who was commissioned to develop the app. The research team engaged with Dementia NI, a membership organization led by people living with dementia to provide support and to influence policy, practice and service delivery. With the support of Dementia NI, we established a User Development Group and this group joined the research team and the app developers for three workshops to inform the design of the app.

## Data Availability

The data that support the findings of this study are available from the corresponding author upon reasonable request.
